# Versatility of the Peroneus Brevis Muscle Flap for Distal Leg, Ankle, and Foot Defects: A Comprehensive Review

**DOI:** 10.1016/j.jpra.2024.06.008

**Published:** 2024-06-18

**Authors:** Vladimir Mégevand, Matteo Scampa, Domizio Suva, Daniel F. Kalbermatten, Carlo M. Oranges

**Affiliations:** a Department of Plastic, Reconstructive and Aesthetic Surgery, Geneva University Hospitals, Geneva University, 1205 Geneva, Switzerland; b Department of Plastic Surgery, Guy's and St Thomas' NHS Foundation Trust, St Thomas' Hospital, London, United Kingdom; c Department of Orthopaedic Surgery, Bone Infection Unit, Geneva University Hospitals, Geneva University, 1205 Geneva, Switzerland

**Keywords:** Peroneus Brevis Flap, Muscle Flap, Local Flap, Distal Leg Reconstruction, Orthoplastics, Comprehensive Review

## Abstract

Soft tissue defects of the distal third of the leg are challenging and management with simple split thickness skin graft or conservative measures is often difficult. The peroneus brevis muscle flap is well described in the literature to cover such defects. The aim of our study was to review the different applications and potential complications of the peroneus brevis muscle flap.

A comprehensive review of all existing evidence on the use of peroneus brevis muscle flaps for coverage of defects in the distal third of the leg in adult populations was performed.

Two hundred forty-eight records were identified in the literature search, among which 15 met the PICOS (Patient, Intervention, Comparison, Outcome and Study design) criteria. All selected studies were retrospective. Overall, 222 patients who received peroneus brevis muscle flaps were analyzed. Indications for reconstruction were post-traumatic defects, infected wounds, and chronic wounds. The overall complication rate was 21% (46/222) with the most commonly reported complication being skin graft loss. We observed 2 cases of partial flap loss, 17 cases of skin graft loss, 2 cases of post-operative hematoma, 2 cases of recurrent infection, 12 cases of partial flap necrosis, 3 cases of skin graft necrosis, and 8 cases of delayed wound healing. Overall, 16 patients (7%) required revision surgery. No cases of donor site morbidity were described.

The current review shows that the peroneus brevis muscle flap is a versatile and reliable option for the coverage of small to medium sized defects of the distal leg, ankle, and foot with low complication rates and donor site morbidity.

## Introduction

Defects around the distal lower leg, lateral malleolus, Achilles tendon, and dorsum of the foot pose unique challenges. Therefore, reconstructive options are often limited due to the paucity of local tissue availability, lack of recipient vessels for free flap transfer, and high risk of bone, tendons, and implant exposure.^1^ These areas are critical for weight-bearing, ambulation, and also aesthetically significant, making the restoration of form and function paramount. In the pursuit of effective solutions, the peroneus brevis (PB) muscle flap has emerged as a promising technique, offering a versatile and anatomically sound option with acceptable outcomes for addressing small to medium size defects of the lower leg, ankle, and foot.

PB is a short muscle that together with peroneus longus comprises the lateral compartment of the lower leg. It arises from the middle and lower thirds of the lateral aspect of the fibula and anterior intermuscular septum. Distally, its tendon runs posteriorly to the lateral malleolus usually in a common synovial sheath together with the peroneus longus and inserts at the base of the fifth metatarsal bone. Motor innervation of the muscle is provided by a motor branch of the superficial peroneal nerve and its main source of blood supply is the anterior tibial artery. Rarely, blood supply is provided by the fibular artery. Distal perforators are located 6-8 cm proximally to the tip of the lateral malleolus.^2^^,^^3^ The flap is raised based on the distal perforators of the peroneal artery and covered with split thickness skin graft (Figure 1a,1b, and 1c). The main function of PB is to evert the foot at the subtalar joint. It is also responsible for plantarflexion of the foot at the ankle; however, the loss of PB with a functional peroneus longus does not appear to cause ankle instability.^4^ Moreover, PB muscle flaps tend to become less bulky over time owing to auto-thinning, thereby, offering better cosmetic results.^5^ Vascularized bone can also be harvested with the PB muscle to reconstruct bony defects in the distal leg.^6^

**Figure 1a:**
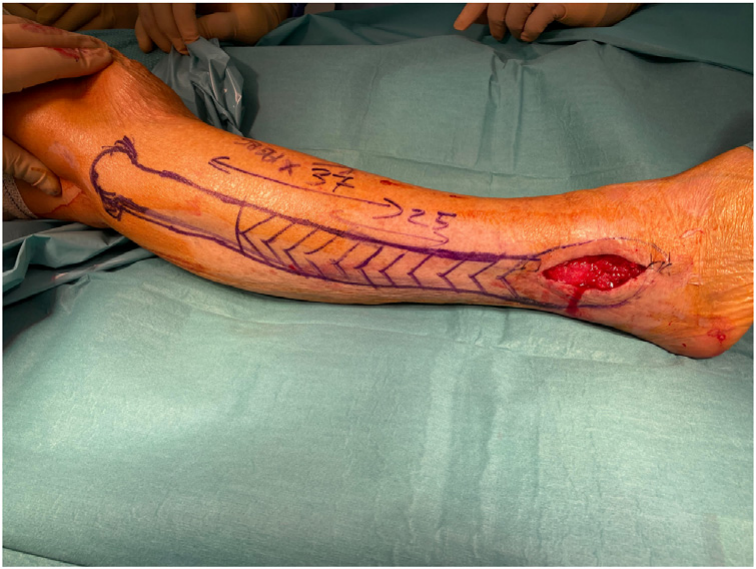
Preoperative markings: The incision is planned 1 cm below the skin projection of the posterior aspect of the fibula in the distal two-thirds of the leg to reconstruct a soft tissue defect of the lateral malleolus.

**Figure 1b:**
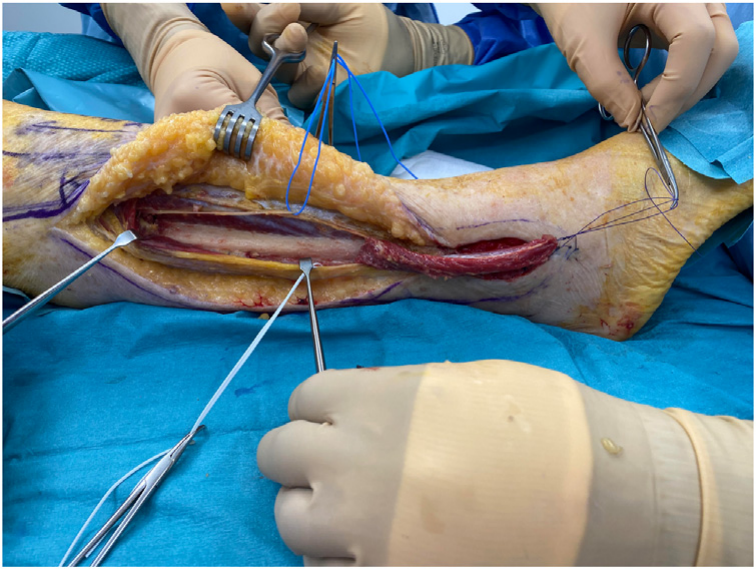
The flap is raised while preserving its vascular pedicle based on the distal perforators emerging from the fibular vessels.

**Figure 1c:**
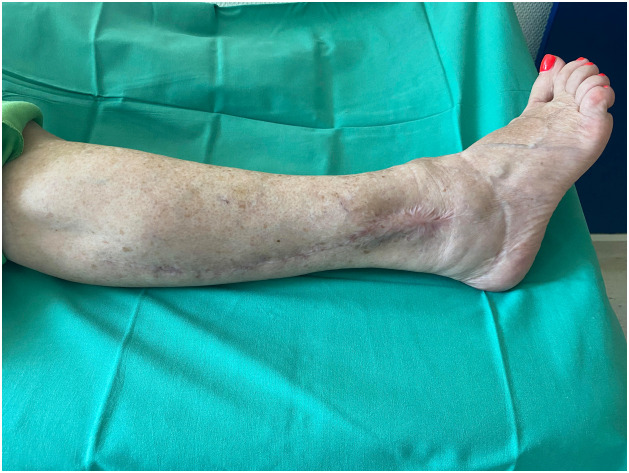
Post-operative picture at the 9-month follow-up.

This review aimed to comprehensively summarize and critically evaluate the existing data on the use of the PB muscle flap, elucidating the indications, outcomes, and potential complications associated with this surgical approach.

## Materials and Methods

### Literature Search Methodology

An exhaustive literature search was performed using PubMed, Cochrane Library, and ResearchGate in April 2023, aimed at all the studies describing the PB muscle flap for distal leg, ankle, and foot soft defect reconstruction. Additional articles were found using the “related articles” feature on PubMed. Inclusion and exclusion criteria were defined using PICOS (Patient, Intervention, Comparison, Outcome and Study design) (Table 1) before conducting the review.

**Table 1:** PICOS for inclusion and exclusion criteria.

	Inclusion	Exclusion
Population	Adult patients who received PB muscle flap to cover distal leg, ankle, and foot defects	Non-adult population, cadaveric studies
Intervention	PB muscle flap to the distal leg with skin graft	Any non-PB flap used to cover distal leg defects
Comparator	Simple skin graft via conservative management	N/A
Outcomes	Major complications requiring revision surgery: flap loss, skin graft loss, infection, and hematoma. Minor complications treated conservatively using partial flap necrosis, skin graft necrosis, and delayed wound healing. Donor site morbidity	N/A
Study design	Retrospective studies	Non-English articles, reviews and meta-analyses, case reports, letters to the editors, and isolated abstracts

PB = peroneus brevis; PICOS = Patient, Intervention, Comparison, Outcome and Study design; N/A = not applicable.

### Selection Process

The titles and abstracts were independently scrutinized by 2 authors (VM and MS) to identify relevant articles for this review, using Rayyan software (Cambridge, Massachusetts, USA).^7^ Any disagreement between reviewers was resolved by consensus after a consultation with a third independent reviewer (CMO). Full-text articles with adult patients benefiting from PB muscle flaps for distal leg, ankle, and foot reconstruction were included and fully read. Non-English articles, meta-analyses, reviews, case reports, letters to the editor, and isolated abstracts were excluded. No limitations were applied to the body mass index or ethnicity of the patients. In addition, the reference lists of all relevant articles were scrutinized to identify additional relevant studies.

### Data Extraction

Data from eligible studies were extracted by 2 authors (VM and MS) using a standardized Excel (Microsoft Corporation, Redmond, Washington, USA) file after reviewing each publication. The following data were collected: study characteristics (first author, publication year, country of origin, study type, study period, total number of patients, mean age, and median follow-up), indication for procedure and post-operative complications. No attempt was made to retrieve missing data from the authors of the included papers.

### Outcome Assessment

All outcomes obtained from the selected studies were reported with the same measurements retrieved from the articles. Post-operative complications were classified into major complications requiring revision surgery (flap loss, skin graft loss, infection, and hematoma) and minor complications treated conservatively (partial flap necrosis, skin graft necrosis, and delayed wound healing). Donor site morbidity was also evaluated in each cohort.

## Results

The literature search yielded 15 relevant articles^5^^,^^8^, ^9^, ^10^, ^11^, ^12^, ^13^, ^14^, ^15^, ^16^, ^17^, ^18^, ^19^, ^20^, ^21^ and sources of information on the applications and potential complications of the PB muscle flap for distal leg, ankle, and foot defects (Table 2).

**Table 2:** Characteristics of the included studies.

Author	Year		Patients	Mean age (y)	F-up (mo)	Defect size (cm^2^)
Jose et al.	2022		6	47	2-27	9.5
Schubert et al.	2021		10	73	/	/
Vaienti et al.	2020		14	54	21	/
Malahias et al.	2020		21	57	9	38.18
Nguyen et al.	2018		17	52	18	13.65
Troisi et al.	2017		11	/	5	31.54
Abd-Al-Moktader	2017		42	40	/	85
Antonini et al.	2017		11	56	40	/
Ceran et al.	2015		17	37	19	12.74
Ng et al.	2010		5	45	/	14.28
Lorenzetti et al.	2009		10	56	29	/
Bach et al.	2007		15	59	9	18.02
Koski et al.	2005		16	55	8	22.4
Lyle et al.	2000		8	52	4-48	21.17
Eren et al.	2000		18	51	24	/

F-up = follow-up; y = years; mo = months; cm^2^ = square centimeters; / = not specified in the selected article.

All studies were of retrospective nature and included a total 222 patients. The size of the included studies ranged from 5 to 42 patients, with a mean follow-up varying from 2 to 48 months after surgery. All studies were published between 2000 and 2022, covering a study period from 1993 to 2019.

The population age was homogenous between the studies with a mean age of 52.5 (15-97; SD 2.9) years, although patients’ age was a limiting factor and 3 studies that were initially selected were removed as they included pediatric patients aged 5, 6, and 7.

The most commonly reported comorbidities were diabetes (63 patients), arterial hypertension (32 patients), the presence of a vascular disease (5 patients), and heavy smoking (36 patients).

The main indications for reconstruction were post-traumatic defects, infected wounds, and chronic wounds.

**Figure 2:**
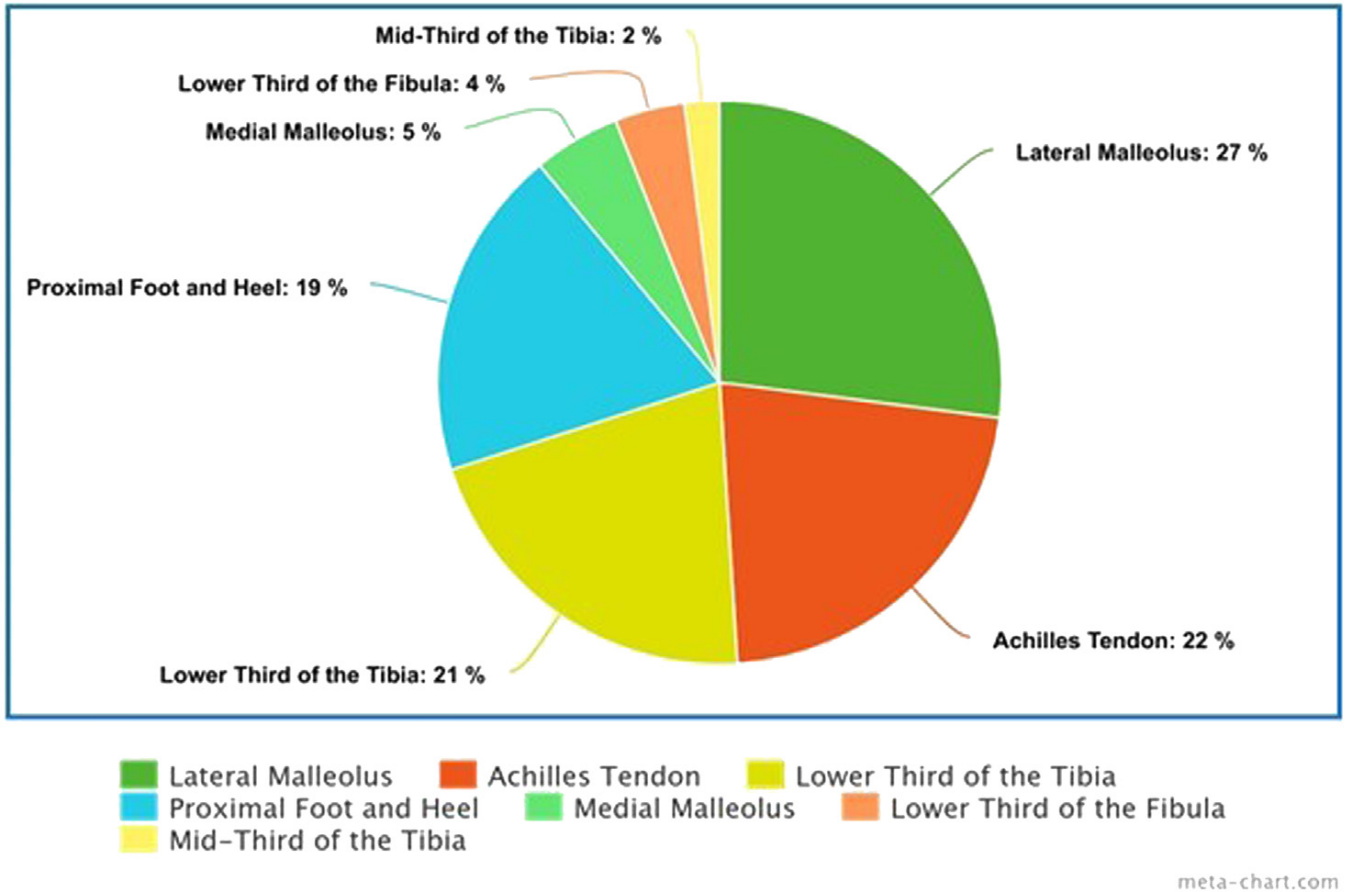
Pie chart representing the location of the defects.

Locations of the defects, classified from most to less frequent, were as follows: the lateral malleolus (n = 59, 27%), Achilles tendon (n = 50, 23%), lower third of the tibia (n = 48, 22%, including 10 on the lateral aspect, 8 on the anterior aspect, 3 on the medial aspect, 3 on the posterior aspect, and 24 of unspecified location), proximal foot and heel (n = 41, 19%), medial malleolus (n = 11, 5%), lower third of the fibula (n =8, 4%), and mid-third of the tibia (n = 5, 2%) (Figure 2).

All defects were considered small to medium size defects, with an average size of 24.18 (SD 4.6) cm^2^.

All flaps performed in the selected articles were strictly muscle flaps and none of the flaps included a skin paddle. Skin graft was performed on the PB muscle flap in all the included articles and the donor site was closed by direct closure.

The overall complication rate was 21% (46/222), including 22 cases of major complications requiring revision surgery and 21 minor complications that were treated conservatively.

Partial flap loss occurred in 2 (1%) patients and none of them required revision surgery. No cases of critical flap loss were reported. Seventeen (8%) patients presented with skin graft loss, 14 of them being partial, and 3 being complete. Post-operative infection was present in 2 (1%) patients. Two (1%) cases of post-operative hematoma were described among all the patients and 1 of them required surgical evacuation.^20^

Partial flap necrosis to the tip occurred in 12 (5%) patients, 2 of them being responsible for skin graft loss; however, no secondary surgery was required. Three (1%) cases of skin graft necrosis were described including 1 complete skin graft necrosis over a completely viable muscle flap.^14^ Finally, 8 (4%) patients presented with a delayed wound healing that was in all cases treated conservatively with dressing changes.

“Partial” flap necrosis and flap loss were defined as necrosis and loss of the distal part (or tip) of the flap, not implying critical damage and total loss of the flap.

No correlation was observed between the size of the defect and any reported complication.

No cases of donor site morbidity were reported in the included articles and all donor sites healed with acceptable scar formation.

## Discussion

Distal leg defects are challenging for plastic surgeons and often require flap coverage due to the paucity of local tissue availability in this area and high risk of bone, tendon, and implant exposure.^1^ Free muscle and fasciocutaneous flaps are often used to cover such defects and have been widely described in the literature. Our recent meta-analysis on the topic^22^ suggested that both flaps can be considered safe and effective for lower legs defects despite fasciocutaneous flaps offering significantly less donor site morbidity, lower risks of infection, and greater aesthetic satisfaction. To cover such defects, free flaps such as the anterior lateral thigh (ALT) flap, gracilis free flap, and medial sural artery perforator (MSAP) free flap are commonly used.^23^^,^^24^ However, microvascular free flaps can be difficult to raise, are time-consuming, need the use of a major vessel of the leg, and rely on the microsurgical expertise of the surgeon.^25^^,^^26^ In addition, free flap transfer in the region of the lateral malleolus is often delicate owing to the paucity of recipient vessels in the area, which are mostly injured during the accident. Local fasciocutaneous flaps are also available, but often lead to bulky results with unaesthetic donor sites.^4^ Muscle flaps are often the first choice when dealing with bone infections associated with soft tissue infections and large defects.^27^, ^28^, ^29^

PB muscle flap can be a versatile option for small to moderate soft tissue defects of the distal leg, foot, and ankle, resisting infection and causing minimum donor site morbidity owing to the maintained ankle mobility provided by the preserved peroneus longus.^4^

By definition, the use of a distally pedicled flap in the lower extremity is not the first line option in the area and free flap transfer is the gold standard.^30^ However, the distally based PB muscle flap has proven to be an effective and reliable regional alternative to cover defects of the distal leg and foot^4^^,^^15^^,^^17^^,^^19^^,^^20^ owing to the microsurgical complexity of free flaps. The use of PB as a proximally based pedicled flap was initially described by Pers and Medgyesi.^31^ It was later described by Eren et al.^17^ as a distally based flap for reconstruction around the ankle.

Among all the included articles, 2 articles^17^^,^^20^ mentioned that patency of the peroneal and posterior tibial arteries was tested using Doppler ultrasound. One study^9^ mentioned that no preoperative assessment using ultrasounds was performed and the remaining 12 studies did not provide details on preoperative assessments for the viability of the muscle prior to the procedure.

The exposure of the bone and metal work in distal leg injuries enhances the risk of infection. One of the fundamental roles of the reconstructive procedure is to prevent bacterial colonization of the area and despite an extensive and radical debridement of the wound prior to coverage, any flap used with this goal has to be well vascularized to allow satisfactory antibiotics diffusion and absorption as well as an efficient immune system response. The rate of post-operative infection was low in our study, occurring only in 2 (0.9%) patients. In one case,^14^ where the flap was used for coverage after debridement of an infection recurrence, a new lesion occurred 6 months post-operatively and eventually required another debridement. In the second case,^17^ preexisting osteomyelitis persisted post-operatively and arthrodesis of the ankle joint was necessary. In both cases, the authors were confident in considering the infection a persistence of poorly treated osteomyelitis rather than a new infection related to the surgery.

Another important factor to be considered is the viability of the selected flap, as necrosis, even if partial, may induce or even worsen the extent of an existing infection. We observed a very low rate of flap necrosis among the patients (5.4%). In contrast, a series led by Barr et al. reported necrosis of the distal tip of the flap in 4 out of 4 (100%) patients.^32^ They stated that the distal segment of the flap was unreliable due to the ligation of the major proximal pedicle in the supposed Type II pattern of vascularization described by Mathes and Nahai.^33^ PB was initially considered a Type II muscle flap but is now considered as Type IV muscle by several authors considering its segmental blood supply. Cadaveric angiography studies have shown that “as long as one pedicle is maintained, complete filling of the muscle can be accomplished.”^34^ This may be due to the poorly described axial vessel running on the posterior aspect of the muscle, linking the perforators.^6^ A hypothetical technical mistake during flap dissection could have likely been the cause of partial flap necrosis in Barr et al.’s series.^32^ Considering the above result and to avoid any technical mistakes, the following principles must be followed perioperatively: the incision line should be marked as a lazy S on the palpable surface of the fibula. The skin flap is raised with the deep fascia while protecting the superficial branch of the peroneal nerve just below the deep fascia and piercing it 15 cm proximally to the lateral malleolus. After identification of the peroneus longus and PB by finding the two tendons distally, they are separated revealing the lateral surface of the fibula and the branches of the peroneal vessels segmentally piercing the muscle are protected. The PB is detached from the anterior intermuscular septum and isolated. It is then elevated, proximally to distally, with the periosteum from the fibula. The branches of the peroneal artery and vein are progressively ligated while the muscle is being raised. The pivotal point of the muscle is located where the large distal vascular branch is found, at approximately two-thirds of the muscle belly (6-8 cm proximally to the tip of the lateral malleolus). The flap is then turned over to cover the defect and a split thickness skin graft is applied on the muscle.

In our study, skin graft loss appears to be the most commonly reported complication affecting 17 (8%) patients with most of them requiring a repeated skin graft. One patient^13^ presented a lack of compliance post-operatively; therefore, no correlation could be made with the surgery itself. Two cases from the same series^14^ reported distal necrosis of the flap. Most authors agree that skin graft loss is due to post-operative oozing and swelling underneath the skin graft owing to the lack of compressive dressings due to fear of flap compression. An alternative to this is to place the skin graft as a second procedure, once the flap is settled well. Negative pressure therapy is commonly used on the muscle flap to effectively secure the skin graft in place, ensuring stability without subjecting the flap to undue pressure.

Flap loss is always a major concern as it can induce further complications including infection and often requires additional procedures. Our study reported 2 cases of partial flap loss and, as expected, correlation with active smoking status and presence of diabetes and arterial hypertension could be made. However, this does not appear to be the only explanation and an anatomical correlation can also be suggested. Previous studies on PB muscle flap found that partial flap loss occurred when the flap was used for medial malleolar defects, possibly due to the arc of rotation required and resulting stretch to the muscle.^34^ Moreover, the study by Bach et al.^20^ reported that 8 out of their 15 patients displayed severe risk factors including the abovementioned ones without specifying the exact number of cases per comorbidity, therefore limiting our data.

Post-operative hematoma was reported in 2 patients overall and 1 of them required surgical evacuation. The authors believe that the likely cause of the hematoma was the dissection of the muscle above the periosteum, initially meant to decrease the risks of heterotrophic ossification over the recipient site but in contrast increased the risks of continuous ooze from the cut end of the muscle fibers leading to hematoma and potential secondary graft loss.^35^ However, there is no evidence supporting the risk of hematoma in older populations receiving the flap and data on any correlation between post-operative bleeding and intake of blood thinners is missing in the included studies.

Overall, 8 patients had delayed wound healing. All of them were managed by daily dressings and healed by secondary intention without further complications or requirement for additional procedures. We believe that the active smoking status and the presence of diabetes observed in most of the affected patients might be the likely cause of poor vascular supply and delay in wound healing.

Another strong point favoring PB muscle flap is the associated low donor site morbidity. There is usually no tension when closing the donor site and direct closure can be performed, whereas other flaps such as the sural flap often require skin grafting on the donor site. Furthermore, harvesting PB muscle flap usually implies no functional impairment allowing ankle stability, foot eversion, and plantar flexion to be well preserved owing to the remaining peroneus longus.^4^ Compared to free flaps, the relatively shorter operative time and absence of microsurgical equipment required for PB muscle flap make it a favorable option. In addition, it is preferred for the coverage of defects in the region of the lateral malleolus where the lack of recipient vessels and venous insufficiency make it a more delicate area for carrying out microvascular free flaps.

The results of this study should be viewed with caution owing to the number of limitations and potential biases influencing these findings. None of the studies included in our review assessed intraoperative venous insufficiency as all the flaps performed were muscle flaps and no skin paddle was included to help assess venous insufficiency. Moreover, the considerable variability in patient follow-up observed in the selected articles introduces bias by affecting the completeness of data. This lack of homogeneity impacted the reliability and comparability of results, making it challenging for us to draw accurate conclusions about the effectiveness or safety of PB muscle flap in the selected population.

## Conclusion

In the ever-evolving landscape of orthoplastic surgery, the PB muscle flap significantly contributes to the surgeon's armamentarium and has emerged as a valuable tool, offering versatile solutions for soft tissue reconstruction of small to medium size defects in the distal leg, ankle, and foot. Through an extensive review of the literature, it became evident that PB flap is an effective and reliable option for addressing various complex defects in the lower extremity. Its ease of harvest, minimal donor site morbidity, and low complication rates make it an attractive choice for primary and salvage reconstruction. Although the PB muscle flaps have shown promising outcomes, it is essential for surgeons to carefully consider patient selection and meticulous surgical techniques to optimize the results.
